# Translocation t(6;7) in AML-M4 cell line GDM-1 results in *MNX1* activation through enhancer-hijacking

**DOI:** 10.1038/s41375-023-01865-5

**Published:** 2023-03-22

**Authors:** Dieter Weichenhan, Anna Riedel, Charlotte Meinen, Alisa Basic, Reka Toth, Marion Bähr, Pavlo Lutsik, Joschka Hey, Etienne Sollier, Umut H. Toprak, Simge Kelekçi, Yu-Yu Lin, Mariam Hakobyan, Aurore Touzart, Ashish Goyal, Justyna A. Wierzbinska, Matthias Schlesner, Frank Westermann, Daniel B. Lipka, Christoph Plass

**Affiliations:** 1 Division of Cancer Epigenomics, German Cancer Research Center (DKFZ), Heidelberg, Germany; 2 Faculty of Biosciences, Ruprecht-Karls-University of Heidelberg, Heidelberg, Germany; 3 Luxembourg Institute of Health, Luxembourg, Luxembourg; 4 Department of Oncology KU Leuven, Leuven, Belgium; 5 Division of Neuroblastoma Genomics, German Cancer Research Center (DKFZ), Heidelberg, Germany; 6 Section of Translational Cancer Epigenomics, Division of Translational Medical Oncology, German Cancer Research Center (DKFZ), National Center for Tumor Diseases (NCT) Heidelberg, Heidelberg, Germany; 7 Université de Paris Cité, Institut Necker Enfants-Malades (INEM), Institut National de la Santé et de la Recherche Médicale (Inserm) U1151, and Laboratory of Onco-Hematology, Assistance Publique-Hôpitaux de Paris, Hôpital Necker Enfants-Malades, Paris, France; 8 Faculty of Applied Informatics, University of Augsburg, Augsburg, Germany; 9 Hopp Children’s Cancer Center Heidelberg (KiTZ), Heidelberg, Germany; 10 German Consortium for Translational Cancer Research (DKTK), Heidelberg, Germany

**Keywords:** Cancer genomics, Cancer models, Acute myeloid leukaemia

## To the Editor:

Recurrent translocations are common in acute myeloid leukemia (AML) and important for cytogenetic classification and prognosis [1]. AML-derived leukemic cell lines have been essential to study molecular defects and to develop novel therapeutic approaches [2, 3]. The leukemic cell line GDM-1 was established from a patient with acute myelomonoblastic leukemia [4]. GDM-1 cells carry a reciprocal translocation t(6;7)(q23;q36) juxtaposing the transcription factor (TF) gene *motor neuron and pancreas homeobox 1* (*MNX1*, also designated *HLXB9* or *HB9*) on chromosome 7 (chr7) to the locus of the transcriptional activator *MYB* on chr6. The translocation does not result in a fusion transcript but leads to aberrant activation of *MNX1*, suspected to be due to altered topologically associating domains, nuclear positioning or ectopic mechanisms [5, 6]. GDM-1 represents the only known AML cell line overexpressing *MNX1*. Here we demonstrate that the interaction between the *MNX1* promoter with a ‘hijacked’ enhancer from the *MYB*/*AHI1* locus leads to ectopic activation of *MNX1*.

**Fig. 1:**
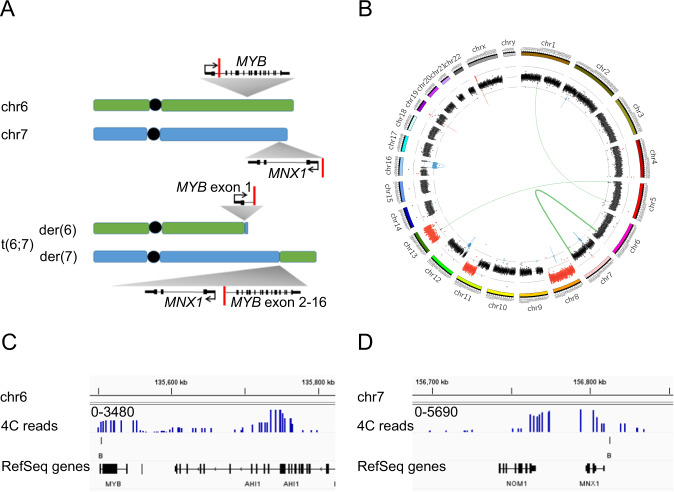
Translocation t(6;7) in GDM-1 juxtaposes *MNX1* with the *MYB*/*AHI1* locus. **A** Schematic overview of chr6 (green), chr7 (blue) and derivative chromosomes der(6) and der(7) resulting from the reciprocal t(6;7) translocation involving *MYB* on chr6 and *MNX1* on chr7. **B** Circos plot showing ten SVs in GDM-1 with the t(6;7) highlighted. **C** Interaction between *MNX1* and the *MYB*/*AHI1* locus indicated by 4C sequence enrichment (upper track) using a 5′ part of *MNX1* as viewpoint (chr7:156800248-156802091). **D** Reciprocal 4C sequence enrichment (upper track) using a region in *MYB* as viewpoint (chr6:135511183-135511908) indicates interaction with the *MNX1* locus. B break point.

We sequenced the whole genome of GDM-1 to identify structural variants (SVs) and mutations in AML-driving genes (Supplementary Table 1). We found two mutations in AML-related genes, one in *PTPN11*, chr12:112888198G>T, hg19; p.A72S, protein ID: Q06124, and one in *CSF1R*, chr5:149441328A>C hg19; Y571D, protein ID P07333. We also found ten SVs including a balanced translocation between chromosomes 6 and 7, t(chr7:156812311;chr6:135505079) and t(chr7:156812323;chr6:135505091) (Fig. 1A, B), which confirms the previously described genomic rearrangement in GDM-1 [4]. The breakpoint (BP) in chr6 resides in intron 1 of the proto-oncogene *MYB*, and the BP in chr7 locates 8954 bp upstream of the transcriptional start site (TSS) of *MNX1* transcript variant 1 (NM_005515.4). In the t(6;7) chromosome, juxtaposed *MNX1* and the rest of *MYB* (exons 2-16) would be transcribed into opposite directions (Fig. 1A) and, hence, exclude the formation of a fusion transcript. Consequently, we neither identified a fusion transcript by transcriptome sequencing nor a MYB/MNX1 fusion protein by Western blot, but we observed instead high expression of *MNX1* at both the transcript and the protein level (Supplementary Fig. 1) with 191.7 transcripts per kilo base million (TPM) in GDM-1 as compared to <3 TPM in 41 unpublished AML cases without *MNX1*-associated rearrangements.

To unravel the mechanism of ectopic *MNX1* activation in GDM-1, we tested by circular chromosome conformation capture (4C), if this activation results from the interaction of the *MNX1* promoter with a hematopoietic enhancer located in or close to the translocated *MYB* locus. For 4C, we used two different viewpoints from the 5′ part of *MNX1* (Supplementary Table 2) and identified two predominant interacting regions on chr6, one covering the *MYB* locus and one covering part of the adjacent *AHI1* gene (Fig. 1C and data not shown). Conversely, reciprocal 4C with the interacting region of *MYB* as viewpoint (Supplementary Table 2) indicated predominant enrichment at *MNX1* and the vicinal *NOM1* gene (Fig. 1D). We did not observe, however, reciprocal interaction between the prominent interacting *AHI1*-exon 20 region (Supplementary Table 2) and the *MNX1* locus (data not shown). Thus, our reciprocal 4C data indicated chromatin interaction between *MNX1* and *MYB* in GDM-1 and supported our hypothesis of enhancer hijacking as the cause of *MNX1* activation.

**Fig. 2:**
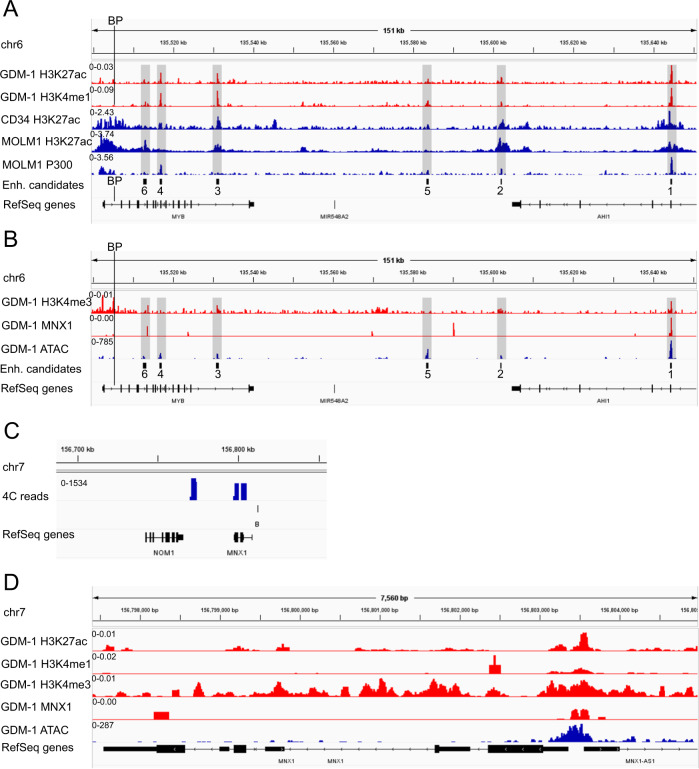
Characteristics of enhancer candidates and chromatin profiles at the *MNX1* locus. **A** Chromatin profiles of the enhancer marks H3K27ac, H3K4me1 (own and public data) and P300 (public data) at the *MYB*/*AHI1* locus. **B** Profiles of promoter mark H3K4me3, MNX1 and open chromatin at the *MYB*/*AHI1* locus. **C** Reciprocal 4C sequence enrichment (upper track) using the E1 region as viewpoint (chr6:135644579-135645062) indicates interaction with the *MNX1* locus. B: break point. **D** Profiles of H3K27ac, H3K4me1, H3K4me3, MNX1 and open chromatin at the *MNX1* locus. In tracks Enh. candidates of (**A**, **B**), numbers refer to the positions of enhancer candidates 1 to 6. BP in (**A**, **B**) indicates the position of the break point in *MYB* of GDM-1.

To identify potential enhancers which might be responsible for *MNX1* activation in GDM-1, we performed Antibody-guided chromatin tagmentation (ACT) followed by sequencing (ACT-seq) to map histone 3 enhancer marks acetylation of lysine 27 (H3K27ac) and mono-methylation of lysine 4 (H3K4me1). To strengthen the ACT-seq data, we further utilized public chromatin immunoprecipitation sequencing (ChIP-seq) profiles, those of H3K27ac in CD34-positive cells and in the chronic myelogenous leukemia cell line MOLM1, and of the H3K27-transacetylase P300, a non-histone enhancer mark, in MOLM1. Based on the chromatin profiles, we selected six enhancer candidates, E1-E6, in the vicinal *MYB* and *AHI1* gene regions distal to the chr6 BP (Fig. 2A). To test the candidate genomic sequences for their enhancer capacity in a luciferase enhancer assay, we cloned them in vector pGL4.23. Three of the six candidates, E1, E4 and E6, proved positive in the enhancer assay (Supplementary Fig. 2, Supplementary Table 3). Among these three, only E1, located in *AHI1*, displayed a peak in all ChIP patterns (see Fig. 2A).

As an additional enhancer property, we profiled DNA methylation associated with enhancer marks H3K27ac and H3K4me1 by combining ACT with subsequent bisulfite treatment and sequencing of the enriched genomic fragments (dubbed ACT-seq-BS), similar to the recently published CUT&Tag-BS [7]. To adjust for different read numbers between the enhancer candidates, we calculated the weighted mean β-values (refer to Supplementary Information). E1 methylation values were lowest, 0 for H3K4me1 and 0.0072 for H3K27ac (Supplementary Fig. 3, Supplementary Table 4). Since the degree of DNA methylation might inversely correlate with enhancer strength [7], E1 might have a stronger effect on *MNX1* activation in GDM-1 than E4 or E6. The contradictory higher strength of E4 compared to E1 in the luciferase enhancer assay might be attributed to the use of Kasumi-1 instead of GDM-1 cells, because the transfection efficiency in GDM-1 was too low to gain suitable luciferase read outs.

We extended chromatin profiling of GDM-1 and mapped promoter mark H3K4me3 as well as targets of MNX1 and open chromatin regions. In the *MYB*/*AHI1* locus, E1 was prominent showing enrichment for all three additional chromatin marks (Fig. 2B). We, thus, performed further 4C using the E1 region which already showed minor interaction with *MNX1* (see Fig. 1C) as viewpoint and found interaction with the *MNX1*/*NOM1* locus (Fig. 2C), similar to the interaction observed in 4C with the *MYB*-associated viewpoint (see Fig. 1D). This reciprocal interaction between *MNX1* and E1 substantiates E1’s role in the activation of *MNX1*.

Inspection of the *MNX1* TSS revealed open chromatin, enrichment of activating marks H3K27ac, H3K4me1 and H3K4me3 as well as weak enrichment of MNX1 itself (Fig. 2D). Thus, we suspect that the chromatin-binding behavior of MNX1 could reflect a feedback loop of MNX1-mediated regulation of its own expression via binding to E1 and to its own promoter. Using the online tool PROMO (http://alggen.lsi.upc.es/cgi-bin/promo_v3/promo/promoinit.cgi?dirDB=TF_8.3), we identified TFs which may cooperate in *MNX1* activation by binding to E1 (Supplementary Table 5): enriched TF binding sites were found for, among others, GATA and C/EBP family members, which are known to be involved in AML pathogenesis [8].

Among 576 MNX1 genomic target sites, commonly found by two independent ACT-seq experiments, we identified 449 in close vicinity (±1.5 kb) to TSS (Supplementary Table 6). This high proportion (80%) of binding close to TSS highlights the prominent role of MNX1 in gene regulation in GDM-1. Metascape analysis with the targeted genes as input revealed their association with chromatin modification, insulin resistance and hematopoiesis as well as the regulation of metabolic processes, signal transduction and response to DNA damage among others (Supplementary Fig. 4). Moreover, MNX1 target genes were found to be involved in diverse malignancies including leukemia and neurological disorders (Supplementary Table 7). To identify TFs potentially cooperating with MNX1 in malignant transformation, we applied HOMER motif analysis to search for enriched sequence motifs among the 576 genomic MNX1 targets and found binding sites of TFs such as NFY, SP and KLF family members (Supplementary Fig. 5), known to be involved in hematopoiesis and leukemic malignancy [9–11].

Our study bears the limitation that we could not functionally prove the requirement of the hematopoietic enhancer E1 to activate *MNX1* in GDM-1. We attempted to generate CRISPR/Cas9-based E1 deletion derivatives of GDM-1 and temporarily found them in bulk, yet, were unable to sustain and expand individual deletion clones. Thus, we suspect that E1 is essential for GDM-1 survival, potentially in regulating *MNX1* as a putative oncogene or as a regulator of MYB function. Future experiments with a tagged version on MNX1 might help to overcome this limitation. Taken together, our data supports the notion of an enhancer hijacking event by which TF MNX1 is ectopically activated in the AML cell line GDM-1, and we propose a hematopoietic enhancer, E1, located in *AHI1*, as being responsible for *MNX1* activation. Binding of MNX1 to this enhancer suggests a feedback loop by which MNX1 perpetuates its own expression together with hematopoietic, immunomodulatory and leukemogenic TFs. Our findings in GDM-1 generate a new mechanistic basis to explain the ectopic expression of MNX1 as, for example, seen in t(7;12) pediatric AML [6, 12]. Our study adds to the growing literature that enhancer-hijacking in acute leukemia represents an important mechanism for gene deregulation [13], examplarily shown for inv(3)/t(3;3)(q21q26) AML resulting in *MECOM* activation via relocation of the *GATA2* enhancer [14], or for acute leukemias of ambiguous lineage, overexpressing *BCL11B* due to enhancer hijacking events involving enhancers from *CDK6* or *ARID1A* gene regions [15].

## Supplementary information

Combined Suplementary Material

Supplementary Table 5

Supplementary Table 6

## Data Availability

The DNA and RNA datasets generated and analysed during the current study are available under GSE221753 (NCBI tracking system 23567412). Whole genome sequencing data is available under accession number PRJNA924216 in the BioSample data base.

## References

[1] Arber DA, Orazi A, Hasserjian R, Thiele J, Borowitz MJ, Le Beau MM (2016). The 2016 revision to the World Health Organization classification of myeloid neoplasms and acute leukemia. Blood.

[2] Drexler HG, Quentmeier H (2020). The LL-100 Cell Lines Panel: Tool for Molecular Leukemia-Lymphoma Research. Int J Mol Sci.

[3] Nilsson T, Waraky A, Ostlund A, Li S, Staffas A, Asp J (2022). An induced pluripotent stem cell t(7;12)(q36;p13) acute myeloid leukemia model shows high expression of MNX1 and a block in differentiation of the erythroid and megakaryocytic lineages. Int J Cancer.

[4] Ben-Bassat H, Korkesh A, Voss R, Leizerowitz R, Polliack A (1982). Establishment and characterization of a new permanent cell line (GDM-1) from a patient with myelomonoblastic leukemia. Leuk Res.

[5] Nagel S, Kaufmann M, Scherr M, Drexler HG, MacLeod RA (2005). Activation of HLXB9 by juxtaposition with MYB via formation of t(6;7)(q23;q36) in an AML-M4 cell line (GDM-1). Genes Chromosomes Cancer.

[6] Ballabio E, Cantarella CD, Federico C, Di Mare P, Hall G, Harbott J (2009). Ectopic expression of the HLXB9 gene is associated with an altered nuclear position in t(7;12) leukaemias. Leukemia.

[7] Li R, Grimm SA, Wade PA (2021). CUT&Tag-BS for simultaneous profiling of histone modification and DNA methylation with high efficiency and low cost. Cell Rep. Methods.

[8] Mulet-Lazaro R, van Herk S, Erpelinck C, Bindels E, Sanders MA, Vermeulen C (2021). Allele-specific expression of GATA2 due to epigenetic dysregulation in CEBPA double-mutant AML. Blood.

[9] Bungartz G, Land H, Scadden DT, Emerson SG (2012). NF-Y is necessary for hematopoietic stem cell proliferation and survival. Blood.

[10] Li C, Dong L, Su R, Bi Y, Qing Y, Deng X (2020). Homoharringtonine exhibits potent anti-tumor effect and modulates DNA epigenome in acute myeloid leukemia by targeting SP1/TET1/5hmC. Haematologica.

[11] Morris VA, Cummings CL, Korb B, Boaglio S, Oehler VG (2016). Deregulated KLF4 Expression in Myeloid Leukemias Alters Cell Proliferation and Differentiation through MicroRNA and Gene Targets. Mol Cell Biol.

[12] von Bergh AR, van Drunen E, van Wering ER, van Zutven LJ, Hainmann I, Lonnerholm G (2006). High incidence of t(7;12)(q36;p13) in infant AML but not in infant ALL, with a dismal outcome and ectopic expression of HLXB9. Genes Chromosomes Cancer.

[13] Xu J, Song F, Lyu H, Kobayashi M, Zhang B, Zhao Z (2022). Subtype-specific 3D genome alteration in acute myeloid leukaemia. Nature.

[14] Groschel S, Sanders MA, Hoogenboezem R, de Wit E, Bouwman BAM, Erpelinck C (2014). A single oncogenic enhancer rearrangement causes concomitant EVI1 and GATA2 deregulation in leukemia. Cell.

[15] Montefiori LE, Bendig S, Gu Z, Chen X, Polonen P, Ma X (2021). Enhancer Hijacking Drives Oncogenic BCL11B Expression in Lineage-Ambiguous Stem Cell Leukemia. Cancer Discov.

