# Supplementary material for: Translocation t(6;7) in AML-M4 cell line GDM-1 results in MNX1 activation through enhancer-hijacking
Source: Leukemia. 2023 Mar 22;37(5):1147–50. doi: 10.1038/s41375-023-01865-5 (PMC10169647; doi:10.1038/s41375-023-01865-5)
Supplement: Supplementary file 1 — Combined Suplementary Material [file 41375_2023_1865_MOESM1_ESM.pdf]

Suplementary Material of Weichenhan et al. “Translocation t(6;7) in AML-M4 cell line GDM-1 results in *MNX1* activation through enhancer-hijacking”

Titles of Supplementary Figures 1 to 5

Supplementary Fig. 1 Expression of MNX1 in GDM-1. Western blot with GDM-1 protein extract and an antibody against MNX1 (left). For quality control of the extract, the blot was stripped and re-hybridized with an antibody against β-actin (right).

Supplementary Fig. 2 Relative luciferase fluorescence generated by enhancer candidates E1, E4 and E6. ‘a’ and ‘b’ in sample names indicate opposite insert orientations in the test vector. The relative fluorescence of candidates E2, E3 and E5 is not shown, because mean values did not reach that of the empty vector or were higher in only one insert orientation. Height of bar plots indicates mean value derived from three or more biological replicates as indicated in the table below the plots. Error bars indicate standard deviation. p-values were calculated with a one-sided t-test.

Supplementary Fig. 3 DNA methylation profiles of enhancer candidates E1, E4 and E6 targeted for H3K27ac and H3K4me1. Tracks show the chromatin profiles above the corresponding CpG β-values.

Supplementary Fig. 4 Enriched terms of cellular processes and pathways associated with MNX1 target genes.

Supplementary Fig. 5 Enriched sequence motifs in MNX1 genomic target sites. Only the first ranked 1 to 10 are shown.

Material and Methods

Cell line cultivation and authentication

Both AML cell lines, GDM-1 (CVCL_1230) and Kasumi-1 (CVCL_0589), were grown in RPMI 1640 (Gibco, 21875-034) supplemented with 20% fetal bovine serum (FBS; Sigma Aldrich, S0615). Cell line authentication was performed November 2021 (Kasumi-1) and April 2022 (GDM-1), and both cell lines are regularly tested for mycoplasma contamination using a commercial test kit (VenorGeM Classic, Minerva Biolabs, cat.no. 11-1050, Berlin, Germany).

Protein extraction and Western blot

Washed cells were 30 min lysed on ice followed by 5 min at 97 °C in 62.5 mM Tris-HCl pH 6.8, 2% sodium dodecyl sulfate (SDS), 10% glycerol, 1 mM DTT, 1 mM NaVO4, 5 mM NaF supplemented with cOmplete^{TM} protease inhibitor (Roche, cat.no. 11697498001, Mannheim, Germany), Benzonase (Santa Cruz Biotechnology, cat.no. sc-202391, Heidelberg, Germany) and PhosStop (Roche, cat.no. 4906837001). The suspension was centrifuged for 5 min in a benchtop centrifuge, and the protein concentration of the supernatant was determined with the Qubit protein assay kit (Thermo Fisher Scientific, cat.no. Q33212, Karlsruhe, Germany). Thirty µg protein in 4x TruPage LDS sample buffer (Sigma, cat.no. PCG3009-10ML, Hamburg, Germany) were separated on a 4-20% SDS-PAGE gradient (BioRad, cat.no. 4561096, Dreieich, Germany) and subsequently transferred to a polyvinylidene difluoride (PVDF) membrane (Millipore, cat.no. IPVH00010, Darmstadt, Germany). The membrane was probed with a primary antibody against MNX1 (Thermo Fisher Scientific cat.no. A303-183A, RRID:AB_10890538) followed by incubation with a secondary antibody coupled to horseradish peroxidase (Santa Cruz Biotechnology cat.no. sc-2030, RRID:AB_631747). The protein signal was visualized by membrane incubation with the Novex ECL HRP Chemiluminescent Substrate Reagent Kit (Invitrogen Thermo Fisher Scientific, cat.no. WP20005) and an Amersham Imager 680 (GE Healthcare, Munich, Germany). For loading quantity control, the membrane was re-probed with a horseradish peroxidase-coupled antibody against β-actin (Santa Cruz Biotechnology cat.no. sc-47778, RRID:AB_626632).

DNA and RNA isolation, whole genome and transcriptome sequencing

Genomic DNA and total RNA were isolated using the QiAmp Micro kit and the RNeasy Plus Mini kit, respectively (Qiagen, cat.no. 56304 and 74136, respectively, Hilden, Germany). Genomic DNA was sequenced on HiSeq X with 150 bp paired-end and RNA was sequenced on NOVASEQ 6000 with 100 bp paired-end. All sequencing reads pertinent to this study were aligned to the reference genome GRCh37/hg19. Non-synonymous mutations in 52 known AML driver genes (Supplementary Table 1) were called following the GATK best practices with mutect2 (https://www.biorxiv.org/content/10.1101/861054v1) in tumor-only mode1. Variants with a frequency > 10^{-4} in the gnomAD2 database were filtered out; this filter was relaxed to 10^{-3} for DNMT3A, TET2, ASXL1 and TP53, which can be mutated in clonal hematopoiesis and thus found in gnomAD [1]. Copy number alterations (CNAs) were called using Control-FREEC3 [2], and structural variants (SVs) were called using manta4

[3]. Considered were only SVs with a minimum length of 40 kb, a minimum of 5 split-reads and 5 spanning pairs supporting the SVs. In addition, pairs of SVs resulting in small insertions or with PolyA insertions were filtered out, because they are most likely the result of retrotransposons which were present in the germline. Moreover, samples from the Simons Genome Diversity Project5 [4] were used to build a panel of controls and filter out putative germline CNAs and SVs. Genomic plots were generated using the circos software6 [5].

Circular chromosome conformation capture (4C)

Circular chromosome conformation capture (4C) was done essentially as described [6] using two million cells and two rounds of restriction digestion, either with *Bgl*II and *Nla*III for the *MNX1*- and *MYB*-associated viewpoints (one and three replicates, respectively) or with *Nla*III and *Csp*6I for the *AHI1*-associated viewpoints (each one replicate), each time followed by ligation. For an additional *MNX1* viewpoint located in exon 1 of variant 1, *Nla*III and *Csp*6I were used. 4C sequencing libraries were generated in two PCR steps. In the first PCR step, the second ligation products, inverse primers (Supplementary Table 2) and Q5 high fidelity enzyme (New England Biolabs, cat.no. M0491, Frankfurt a.M., Germany) were used. Reaction conditions were 98°C for 30 sec, 10 cycles with 98°C for 15 sec, 61°C, 60°C or 54°C depending on the viewpoint for 20 sec with 0.5°C touch-down per cycle, 72°C for 2 min, then 25 or 30 cycles with 98°C for 15 sec, 56°C, 55°C or 49°C, depending on the viewpoint (see Supplementary Table 2), for 20 sec, 72°C for 2 min, finally followed by 72°C for 1 min. PCR products were purified with HighPrep beads (Biozym, cat.no. 220002, Hessisch Oldendorf, Germany), and DNA concentrations were determined with the Qubit dsDNA HS Assay (Thermo Fisher Scientific, cat.no. Q32854). Libraries were generated in the second PCR step with about 5 ng DNA from the first PCR step essentially according to [7] under real-time conditions with a Lightcycler 480 (Roche) and 25 µl reaction volumes using Kapa 2G Robust Hot Start ReadyMix (Merck, cat.no. KK5702, Darmstadt, Germany), 95°C, 3 min (initial melting) and 95°C, 20 sec, 62°C, 15 sec, 72°C, 40 sec (cycling). Library DNA was bead-purified, concentration was determined with the Qubit dsDNA HS Assay and fragment size was determined with a TapeStation 4150 with D1000 High Sensitivity Assay (Agilent, cat.no. 5067- 5585, Waldbronn, Germany). For sequencing, differently barcoded libraries were pooled in equimolar ratios.

Dual luciferase/renilla reporter assays

The six putative enhancers in the *MYB*/*AHI1* locus were PCR-amplified from commercial human DNA (Roche, cat.no. 11691112001) using Q5 DNA polymerase (New England Bioloabs, cat.no. M0491), cloned into reporter vector pGL4.23 (Promega, cat.no. E841A,

Heidelberg, Germany) in both orientations and sequence verified by Sanger sequencing (GATC Biotech, Ebersberg, Germany). About 900,000 Kasumi-1 cells were transfected using the TransIT LT1 transfection reagent (Mirus, cat.no. MIR 2304, by VWR International, Darmstadt, Germany) with 1.2 µg plasmid mix consisting of 480 ng of the reporter construct or empty vector pGL4.23, 240 ng of the pRL-TK Renilla luciferase reporter vector (Promega, cat.no. E2241) and, as stuffer DNA, 20 ng of a pGL3 (Promega, cat.no. E1751) deletion derivative with a non-functional luciferase gene. The dual luciferase/renilla readout was performed 48 h after transfection using the SpectraMax M5 (Molecular Devices, Wokingham, UK), and luciferase signals were normalized to those from renilla. From each transfection, mean values of four to six technical replicates were determined; per construct, three biological replicates were analyzed.

Mean value barplots with standard deviations and single values of biological replicates were generated using the R statistical environment, version 3.5.3. Statistical evaluation of differences between mean values and empty vector (values of normalized signals set to 1) was performed by one-sample t-test.

Antibody-guided Chromatin Tagmentation (ACT-seq)

Genome-wide targeting of histone modifications and transcription factor (TF) MNX1 was done by ACT-seq, largely according to [8]. The pA-Tn5ase protein was isolated from E. coli (C3013, New England Biolabs) transformed with plasmid pET15bpATnp (Addgene, cat.no. 121137, Watertown, MA, USA). The pA-Tn5 transposome (pA-Tn5ome) was generated by mixing pA-Tn5ase (final concentration either 1.9 µM or 3.3 µM, depending on the pA-Tn5ase preparation) and Tn5ME-A+B load adaptor mix (final concentration 3.3 µM) in complex formation buffer (CB; [8]). The pA-Tn5ome-antibody (pA-Tn5ome-ab) complexes were formed by mixing 1 µl pA-Tn5ome with 0.8 µl CB and 0.8 µl antibody solution. We used antibodies against histone H3K27ac (Abcam cat.no. ab4729, RRID:AB_2118291 and Abcam cat.no. ab177178, RRID:AB_2828007, Cambridge, UK), H3K4me1 (Abcam cat.no. ab8895, RRID:AB_306847), H3K4me3 (Diagenode, cat.no. C15410003, RRID:AB_2924768, Liege, Belgium), H2B (Hölzel Diagnostika, cat.no. M30930, RRID:AB_2924769, Cologne, Germany), IgG (Millipore cat.no. PP64, RRID:AB_97852) and MNX1 (Thermo Fisher Scientific cat.no. PA5-23407, RRID:AB_2540929). For pA-Tn5ome-ab complex binding and tagmentation, 200000 (for MNX1/pMNX1 targeting) or 50000 (all other targets) cells were used. For normalization of sequence reads between biological replicates, ~4,000 permeabilized nuclei of yeast *Saccharomyces cerevisiae*, prepared according to [9] and incubated with pA-Tn5ome-ab complex targeting yeast H2B, were spiked into each mix of cells and pA-Tn5ome-ab complex. Tagmented DNA was purified with a MinElute kit (Qiagen,

cat.no. 28004) and eluted with 20 µl elution buffer (EB). Sequencing libraries were generated under real-time conditions with a LightCycler 480 in 50 µl reaction mixes consisting of 20 µl tagmented DNA eluate, 25 µl NEBNext High Fidelity 2X Mix (New England Biolabs, cat.no. M0541), 0.5 µl 100xSYBRGreen, 2.5 µl primer Tn5McP1n and 2.5 µl barcode primer [7]. Reaction conditions were 72°C, 5 min (gap repair); 98°C, 30 sec (initial melting); 98°C, 10 sec, 63°C, 10 sec, 72°C, 10 sec (cycling). Cycling was stopped when the increase of fluorescence units (FUs) was 5 or higher. Libraries were purified with HighPrep beads with a bead:DNA ratio of 1.4:1 and 12 µl EB. Quantity and fragment size of the libraries were determined with a Qubit dsDNA HS assay kit and a TapeStation 4150 with D1000 High Sensitivity Assay, respectively. Six to eight differently barcoded libraries were multiplexed and sequenced on a single lane of a NextSeq 550 system (paired-end, 75 bp) with mid-output at the Genome and Proteome Core Facility of the DKFZ. All ACT-seq experiments were done in at least three replicates.

ACT with subsequent bisulfite treatment of the enriched genomic fragments (ACT-seq-BS)

ACT-seq-BS was done by combining ACT-seq and tagmentation-based whole genome bisulfite sequencing (TWGBS) [7] as follows. For the transposome, a methylated load adapter consisting of the oligonucleotides
5'-T[5mC]GT[5mC]GG[5mC]AG[5mC]GT[5mC]AGATGTGTATAAGAGA[5mC]AG-3'
and 5'-[Phos]-CTGTCTCTTATACA[ddC]-3' was generated. The antibody-transposome complex was generated with antibodies against H3K27ac and H3K4me1 as described above. No yeast nuclei spike-in was used. Tagmentation reaction and DNA purification were followed by oligonucleotide replacement/gap repair (methylated replacement oligonucleotide
5'-[Phos]-[5mC]TGT[5mC]T[5mC]TTATA[5mC]A[5mC]AT[5mC]T[5mC][5mC]GAG
[5mC][5mC][5mC]A[5mC]GAGA[5mC] [inv dT]-3') and bisulfite conversion as described previously for TWGBS. Sequencing library generation and all other subsequent steps were performed as described in the chapter above. Each ACT-seq-BS experiment was done in two replicates.

Assay for transposase-accessible chromatin by sequencing (ATAC-seq)

ATAC-seq was done essentially as described by Corces et al. [10] in four replicates using about 50,000 cells and the Nextera DNA library prep kit (Illumina, cat.no. 15028212, Berlin, Germany). Libraries were generated as described for ACT-seq, but cycling conditions were 98°C, 10 sec, 63°C, 30 sec, 72°C, 30 sec. Library DNA purification and quality assessment was done as described for ACT-seq.

Sequencing of ACT-seq, ACT-seq-BS and ATAC-seq library pools and data analysis

ACT-seq, ACT-seq-BS and ATAC-seq library pools were sequenced on an Illumina NextSeq 550 instrument with PE, 75 bp, mid-output mode. 4C library pools were sequenced on an Illumina HiSeq 2000 instrument with V4, PE, 125 bp mode. Upstream processing of ATAC-seq and ACT-seq data was performed as follows. TrimGalore v. 0.4.4 (https://www.bioinformatics.babraham.ac.uk/projects/trim_galore) was deployed together with Cutadapt v. 1.14 (https://cutadapt.readthedocs.io/en/stable/) using the non-default parameters "--paired", "--nextera", "--length_1 35", and "--length_2 35" to perform adapter and quality trimming. Bowtie2 v. 2.2.6 [11] was used with the "--very-sensitive" flag and a maximum insertion length of 2500 bp to map trimmed reads against the GRCh37/hg19 reference genome. Aligned reads belonging to the same lane-multiplexed library were combined using SAMtools merge v. 1.5 (https://github.com/samtools/samtools/releases/tag/1.5). PCR duplicates were removed by means of Picard MarkDuplicates v. 2.17.4 (https://broadinstitute.github.io/picard/) for ATAC-seq but not ACT-seq data. Discordant mappings and alignments with a Phred score below 20 were removed using SAMtools view. As Adey et al. [12] demonstrated that fragments resulting from tagmentation cannot be smaller than 38 bp, all alignments corresponding to fragments sizes below that threshold were discarded. The ends of reads were adjusted to represent the center of the transposition event. For ACT-seq, trimmed reads were additionally aligned against the *S. cerevisiae* R64 reference genome followed by post-alignment filtering as described above. To derive a library-specific scaling factor, the multiplicative inverse of the number of filtered alignments against the yeast genome were calculated. Owing to this normalization, the signal ranges in bigwig-files and IGV-browser tracks are close to zero. Coverage tracks were generated using the bamCoverage functionality of Deeptools v. 3.1.1 (https://anaconda.org/bioconda/deeptools/files?version=3.1.1) with the non-default parameters "--ignoreForNormalization chrM chrY chrX" and "--effectiveGenomeSize 2652783500" as well as the "--scaleRatio" option to specify the spike-in-derived scaling factor. For ACT-seq-BS, the aligner and methylation caller Bismark (version 0.22.3) [13] was used to obtain the methylation levels. The Bismark pipeline involves four steps. First, adapters were trimmed, and read quality was controlled by TrimGalore (version 0.6.7; https://www.bioinformatics.babraham.ac.uk/projects/trim_galore). Second, clean reads were aligned back to the human genome (GRCh37/hg19) by Bismark. Third, duplicated reads were removed by the deduplicate_bismark function. Fourth, methylation was called by the bismark_methylation_extractor function. Finally, methrix (version 1.2.06) [14] was adopted to

produce the collapsed BED files for the CpG sites with the called methylation levels (β-value) and supporting read coverage. To adjust for different read numbers between the enhancer candidates, the weighted mean β-values which consider the read coverage (Cov) were calculated according to the formula

β-value = (Cov_CpG1 * beta_CpG1 + Cov_CpG2 * beta_CpG2 +…..) / (Cov_CpG1 + Cov_CpG2 + ….). The ATAC-seq accessibility signal was smoothed by centering a 73 bp window on the transposition event's midpoint of each read using a custom script; the resulting tag coordinates were used for all downstream analyses. The analysis procedures were implemented as fully containerized workflows using the Common Workflow Language v. 1.0 (https://www.commonwl.org/v1.0/Workflow.html). Genomic targets of MNX1 were identified from overlapping peak coordinates of two independent ACT-seq experiments. Peak calling was performed with MACS v.2.2.6 (https://pypi.org/project/MACS2/) using a wrapper script callpeaks provided by Encode (https://www.encodeproject.org/chip-seq/histone/); for input subtraction, ACT-seq data from non-targeting IgG control was used. Peak coordinates were overlapped using intersectBed (vers. 2.26.0; https://bedtools.readthedocs.io/en/latest/content/tools/intersect.html) with default parameters. Common MNX1 peaks were annotated to hg19 with Homer annotatePeaks.pl using default settings (http://homer.ucsd.edu/homer/ngs/annotation.html). When a common MNX1 peak located +/- 1.5 kb vicinal to a TSS, the corresponding gene was considered MNX1 target gene. 4C-seq data processing and analysis was done according to [15] with pipe4C using single reads starting with the *Bgl*II (*MNX1*- and *MYB*-associated viewpoints) or the *Nla*III (*MNX1*- and *AHI1*-associated viewpoints) restriction site; the pipe4C pipeline was applied with default parameters under R3.6.2.

Sources of public ChIP data and search tools

Public ChIP data of human mobilized CD34 cells profiled for H3H27ac were obtained from https://www.ncbi.nlm.nih.gov/gds/?term=GSM772885 and of MOLM1 cells profiled for H3K27ac and P300 from [16]. The search for TF binding sites in enhancer candidate E1 was done with the PROMO online search tool (http://alggen.lsi.upc.es/cgi-bin/promo_v3/promo/promoinit.cgi?dirDB=TF_8.3). Enriched TF binding sites among 576 MNX1 target regions were identified by Homer motif search (http://homer.ucsd.edu/homer/ngs/peakMotifs.html) with default parameters. The search for enriched cellular processes and disease genes was done using the online tool Metascape (https://metascape.org/).

References

1. Karczewski KJ, Francioli LC, Tiao G, Cummings BB, Alfoldi J, Wang Q*, et al.* The mutational constraint spectrum quantified from variation in 141,456 humans. Nature 2020; 581(7809)**:** 434-443.

2. Boeva V, Popova T, Bleakley K, Chiche P, Cappo J, Schleiermacher G*, et al.* Control-FREEC: a tool for assessing copy number and allelic content using next-generation sequencing data. Bioinformatics 2012; 28(3)**:** 423-425.

3. Chen X, Schulz-Trieglaff O, Shaw R, Barnes B, Schlesinger F, Kallberg M*, et al.* Manta: rapid detection of structural variants and indels for germline and cancer sequencing applications. Bioinformatics 2016; 32(8)**:** 1220-1222.

4. Mallick S, Li H, Lipson M, Mathieson I, Gymrek M, Racimo F*, et al.* The Simons Genome Diversity Project: 300 genomes from 142 diverse populations. Nature 2016; 538(7624)**:** 201-206.

5. Krzywinski M, Schein J, Birol I, Connors J, Gascoyne R, Horsman D*, et al.* Circos: an information aesthetic for comparative genomics. Genome Res 2009; 19(9)**:** 1639-1645.

6. van de Werken HJ, Landan G, Holwerda SJ, Hoichman M, Klous P, Chachik R*, et al.* Robust 4C-seq data analysis to screen for regulatory DNA interactions. Nat Methods 2012; 9(10)**:** 969-972.

7. Weichenhan D, Wang Q, Adey A, Wolf S, Shendure J, Eils R*, et al.* Tagmentation-Based Library Preparation for Low DNA Input Whole Genome Bisulfite Sequencing. Methods Mol Biol 2018; 1708**:** 105-122.

8. Carter B, Ku WL, Kang JY, Hu G, Perrie J, Tang Q*, et al.* Mapping histone modifications in low cell number and single cells using antibody-guided chromatin tagmentation (ACT-seq). Nat Commun 2019; 10(1)**:** 3747.

9. Orsi GA, Kasinathan S, Zentner GE, Henikoff S, Ahmad K. Mapping regulatory factors by immunoprecipitation from native chromatin. Curr Protoc Mol Biol 2015; 110**:** 21 31 21-21 31 25.

10. Corces MR, Trevino AE, Hamilton EG, Greenside PG, Sinnott-Armstrong NA, Vesuna S*, et al.* An improved ATAC-seq protocol reduces background and enables interrogation of frozen tissues. Nat Methods 2017; 14(10)**:** 959-962.

11. Langmead B, Salzberg SL. Fast gapped-read alignment with Bowtie 2. Nat Methods 2012; 9(4)**:** 357-359.

12. Adey A, Morrison HG, Asan, Xun X, Kitzman JO, Turner EH, *et al.* Rapid, low-input, low-bias construction of shotgun fragment libraries by high-density in vitro transposition. Genome Biol 2010; 11(12)**:** R119.

13. Krueger F, Andrews SR. Bismark: a flexible aligner and methylation caller for Bisulfite-Seq applications. Bioinformatics 2011; 27(11)**:** 1571-1572.

14. Mayakonda A, Schonung M, Hey J, Batra RN, Feuerstein-Akgoz C, Kohler K*, et al.* Methrix: an R/bioconductor package for systematic aggregation and analysis of bisulfite sequencing data. Bioinformatics 2020; 36: 5524–5525.

15. Krijger PHL, Geeven G, Bianchi V, Hilvering CRE, de Laat W. 4C-seq from beginning to end: A detailed protocol for sample preparation and data analysis. Methods 2020; 170**:** 17-32.

16. Groschel S, Sanders MA, Hoogenboezem R, de Wit E, Bouwman BAM, Erpelinck C*, et al.* A single oncogenic enhancer rearrangement causes concomitant EVI1 and GATA2 deregulation in leukemia. Cell 2014; 157(2)**:** 369-381.

Supplementary Fig. 1

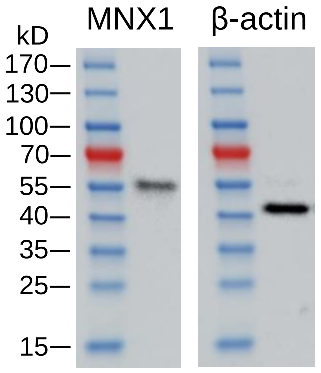

Supplementary Fig. 2

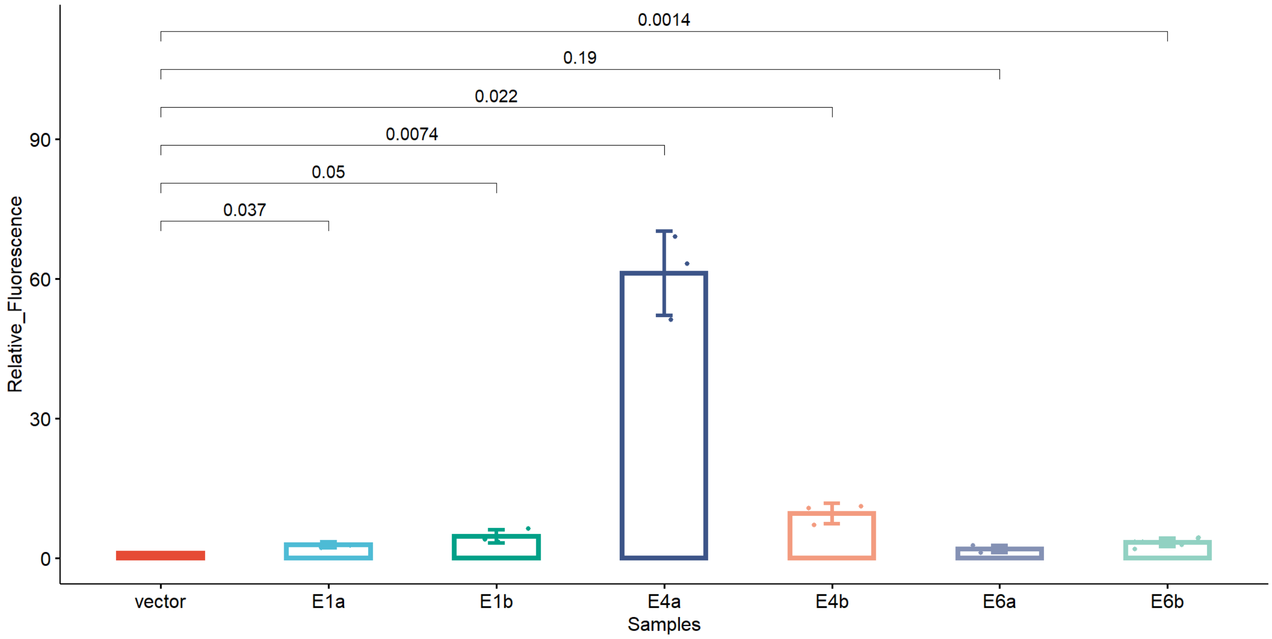

Supplementary Fig. 3

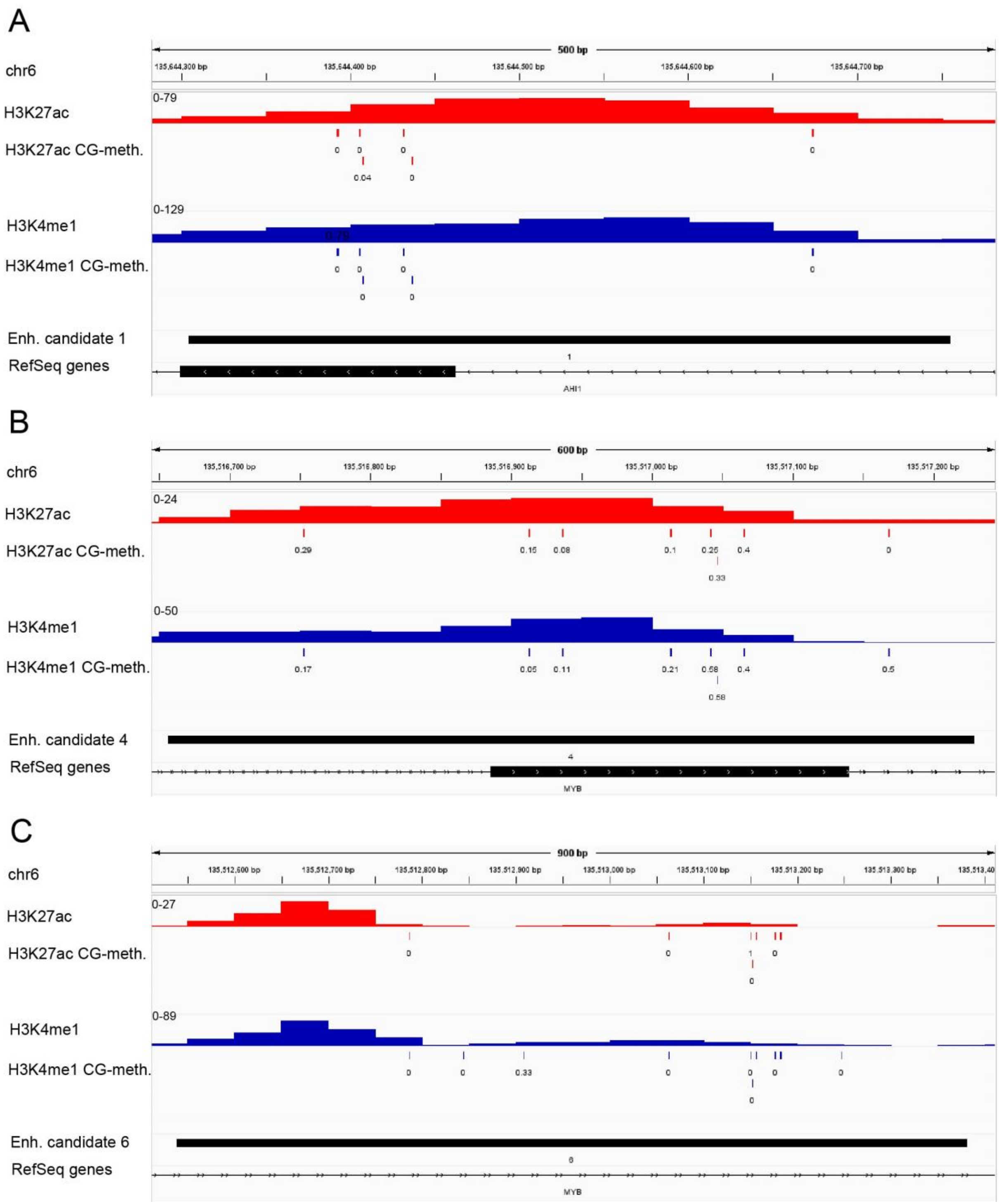

Supplementary Fig. 4

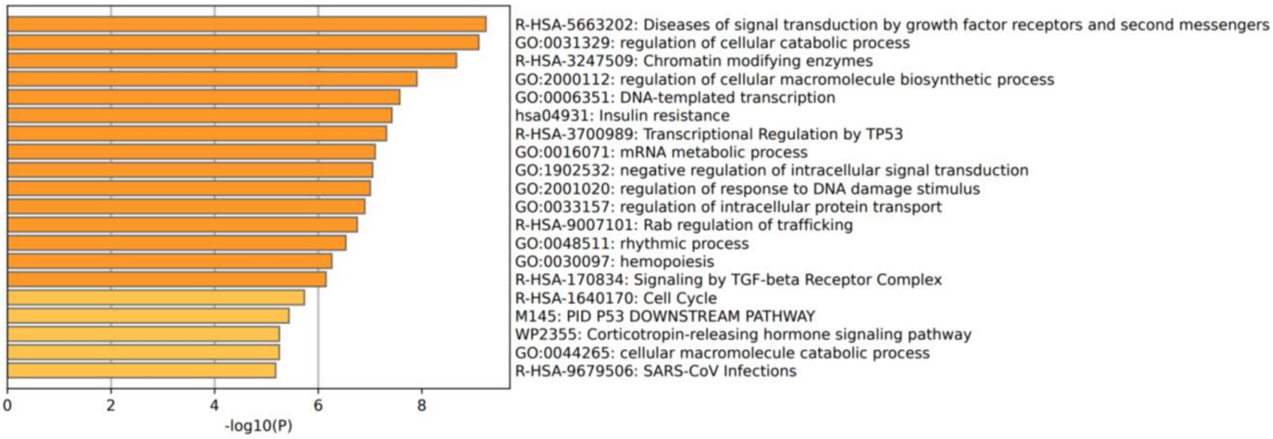

Supplementary Fig. 5

Rank	Motif	Name	P-value	log P-pvalue	q-value (Benjamini)
1	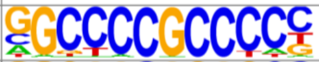	Sp1(Zf)/Promoter/Homer	1e-64	-1.486e+02	0.0000
2	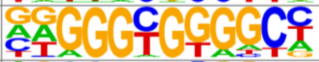	KLF1(Zf)/HUDEP2-KLF1-CutnRun(GSE136251)/Homer	1e-57	-1.320e+02	0.0000
3	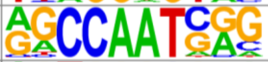	NFY(CCAAT)/Promoter/Homer	1e-53	-1.235e+02	0.0000
4	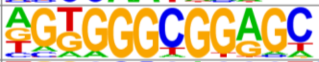	Sp5(Zf)/mES-Sp5.Flag-ChIP-Seq(GSE72989)/Homer	1e-51	-1.190e+02	0.0000
5	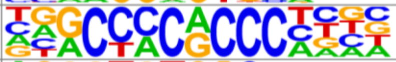	KLF3(Zf)/MEF-Klf3-ChIP-Seq(GSE44748)/Homer	1e-51	-1.186e+02	0.0000
6	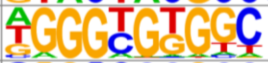	KLF5(Zf)/LoVo-KLF5-ChIP-Seq(GSE49402)/Homer	1e-42	-9.691e+01	0.0000
7	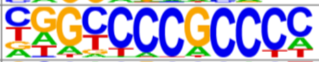	Sp2(Zf)/HEK293-Sp2.eGFP-ChIP-Seq(Encode)/Homer	1e-41	-9.600e+01	0.0000
8	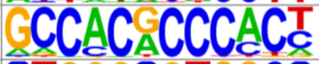	Klf9(Zf)/GBM-Klf9-ChIP-Seq(GSE62211)/Homer	1e-39	-9.020e+01	0.0000
9	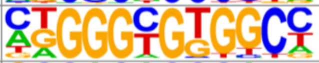	KLF6(Zf)/PDAC-KLF6-ChIP-Seq(GSE64557)/Homer	1e-39	-8.988e+01	0.0000
10	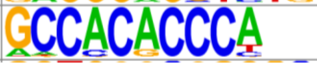	Klf4(Zf)/mES-Klf4-ChIP-Seq(GSE11431)/Homer	1e-32	-7.442e+01	0.0000

Supplementary Table 1. AML driver genes

ASXL1	*SMC1A*
ASXL2	*SMC3*
BCOR	*SRSF2*
CEBPA	*STAG2*
CEBPG	*SUZ12*
CREBBP	*TET1*
CSF1R	*TET2*
DNMT3A	*TP53*
DNMT3B	*U2AF1*
ETV6	*WT1*
EZH2	*ZRSR2*
FLT3	
GATA2	
IDH1	
IDH2	
JAK2	
JARID2	
KAT6A	
KDM3B	
KDM6A	
KIT	
KMT2A	
KMT2C	
KMT2D	
KMT2E	
KRAS	
MED12	
NCOR1	
NCOR2	
NF1	
NOTCH1	
NOTCH2	
NPM1	
NRAS	
NSD1	
PHF6	
PTPN11	
RB1	
RUNX1	
SF3B1	
SMARCA2	
SMARCA4	

Supplementary Table 2. Primers used for 4C

Viewpoint region	Viewpoint primer	Sequence[1]	hg19 primer coordinate	Touchdown PCR	PCR cycles
MNX1 exon 2-intron 2	BglII_4cMNX1F2	TCGTCGGCAGCGTCAGATGTG TATAAGAGACAGTCGGGTTAAT CATTAGATCT	chr7:156802073-156802091		
	NlaIII_4cMNX1R1	GTCTCGTGGGCTCGGAGATGT GTATAAGAGACAGCTGCCTGT AATTTGCGTAAT	chr7:156800248-156800266	54°C-49°C	40
MNX1 exon 1	NlaIII_4cMNX1F2	GTCTCGTGGGCTCGGAGATGT GTATAAGAGACAGACTCAGCC GAGGGTGACCATG	chr7:156803190-156803210		
	Csp6I_4cMNX1R2	TCGTCGGCAGCGTCAGATGTG TATAAGAGACAGAGCACCCGG CGCTCTCCTAC	chr7:156802493-156802512	61°C-56°C	35
MYB intron 4-intron 5	BglII_4CMYBR1	GTCTCGTGGGCTCGGAGATGT GTATAAGAGACAGTCATTCTTC AGTGTTACAAGATCT	chr6:135511182-135511205		
	NlaIII_4CMYBF1	TCGTCGGCAGCGTCAGATGTG TATAAGAGACAGTCAGCTACC ATTTTCCTGATAACC	chr6:135511885-135511908	60°C-55°C	40
AHI1 exon 20-intron20	NlaIII_4cAHI1R1	GTCTCGTGGGCTCGGAGATGT GTATAAGAGACAGTATTTAGGA GAACAAGTAGCCATG	chr6:135748429-135748452		
	Csp6I_4cAHI1F1	TCGTCGGCAGCGTCAGATGTG TATAAGAGACAGGCTTTGGTG AAGGATTAACTTTTCC	chr6:135748845-135748869	61°C-56°C	35
AHI1 enhancer candidate E1	NlaIII_4cAHI1F1	GTCTCGTGGGCTCGGAGATGT GTATAAGAGACAGATTTAAGA GGAAAAGCCTTTTCATG	chr6:135645038-135645062		
	Csp6I_4cAHI1R1	TCGTCGGCAGCGTCAGATGTG TATAAGAGACAGGCAAACCTT GAGACAGCCTCAC	chr6:135644579-135644600	61°C-56°C	35

[1]Genome-specific sequence corresponding to hg19 coordinate is underlined;

break points locate at chr7:156812311 and chr6:135505079

Supplementary Table 3. Relative luciferase fluorescence values of enhancer candidates E1, E4 and E6

hg19 locus	chr6:135644304-135644755		chr6:135516656-135517229		chr6:135512538-135513381	
Candidate[1]	E1a	E1b	E4a	E4b	E6a	E6b
	3.4	6.2	51.3	7	1.2	4.2
	2.7	4	69	11	1.7	1.9
	2.2	3.5	63.2	10.6	2.7	3.5
						4.3
						3.4
						2.8
mean	2.8	4.6	61.2	9.5	1.9	3.4

[1]a and b indicate the alternative orientations of the candidate sequence in the luciferase test vector pGL4.23

Supplementary Table 4. Read numbers and methylation β-values of CpGs covered by enhancer candidates E1, E4 and E6 targeted for H3K27ac and H3K4me1

Enhancer candidate E1	H3K27ac		H3K4me1	
CpG position in chr6	reads	β-value	reads	β-value
135644392	22	0	43	0
135644405	26	0	51	0
135644407	25	0.04	51	0
135644431	28	0	43	0
135644436	28	0	41	0
135644673	10	0	26	0
Weighted mean β-value		0.0072		0

Enhancer candidate E4	H3K27ac		H3K4me1	
CpG position in chr6	reads	β-value	reads	β-value
135516752	7	0.29	12	0.17
135516912	13	0.15	21	0.05
135516936	12	0.08	28	0.11
135517013	10	0.1	14	0.21
135517041	8	0.25	12	0.58
135517046	6	0.33	12	0.58
135517065	5	0.4	5	0.4
135517168	1	0	2	0.5
Weighted mean β-value		0.1923		0.2456

Enhancer candidate E6	H3K27ac		H3K4me1	
CpG position in chr6	reads	β-value	reads	β-value
135512786	1	0	5	0
135512844			2	0
135512908			6	0.33
135513063	1	0	9	0
135513150	2	1	5	0
135513152	2	0	4	0
135513156	2	0	4	0
135513176	1	0	4	0
135513182	1	0	3	0
135513247			3	0
Weighted mean β-value		0.2000		0.0440

Supplementary Table 7. Enrichment in DisGeNET, a platform integrating information on human disease-associated genes					
**GO**	**Description**	**Count**	**%**	**Log10(P)**	**Log10(q)**
C1328504	Hormone refractory prostate cancer	29	6.9	-6.9	-3.7
C0240635	Byzanthine arch palate	24	5.7	-6.8	-3.7
C0349604	Intracranial Meningioma	7	1.7	-6.1	-3.2
C0524587	Mean Corpuscular Volume (result)	16	3.8	-5.9	-3
C0232466	Feeding difficulties	21	5	-5.4	-2.6
C0427144	Toe-walking gait	7	1.7	-5.2	-2.5
C3887461	Head and Neck Carcinoma	28	6.7	-5.2	-2.4
C0020224	Polyhydramnios	13	3.1	-5.1	-2.4
C0856863	Broad-based gait	8	1.9	-5	-2.3
C0278996	Malignant Head and Neck Neoplasm	27	6.4	-4.9	-2.3
C0282160	Aplasia Cutis Congenita	18	4.3	-4.9	-2.3
C0023492	Leukemia, T-Cell	19	4.5	-4.6	-2
C0037772	Spastic Paraplegia	10	2.4	-4.4	-1.9
C1708349	Hereditary Diffuse Gastric Cancer	15	3.6	-4.4	-1.9
C0011168	Deglutition Disorders	17	4	-4.4	-1.9
C0085669	Acute leukemia	23	5.5	-4.4	-1.9
C0037579	Soft Tissue Neoplasms	10	2.4	-4.4	-1.9
C0272386	Hypertrophy of tonsils	4	0.95	-4.3	-1.8
C0007786	Brain Ischemia	16	3.8	-4.3	-1.8
C4025249	Abnormality of the intervertebral disk	5	1.2	-4.3	-1.8
